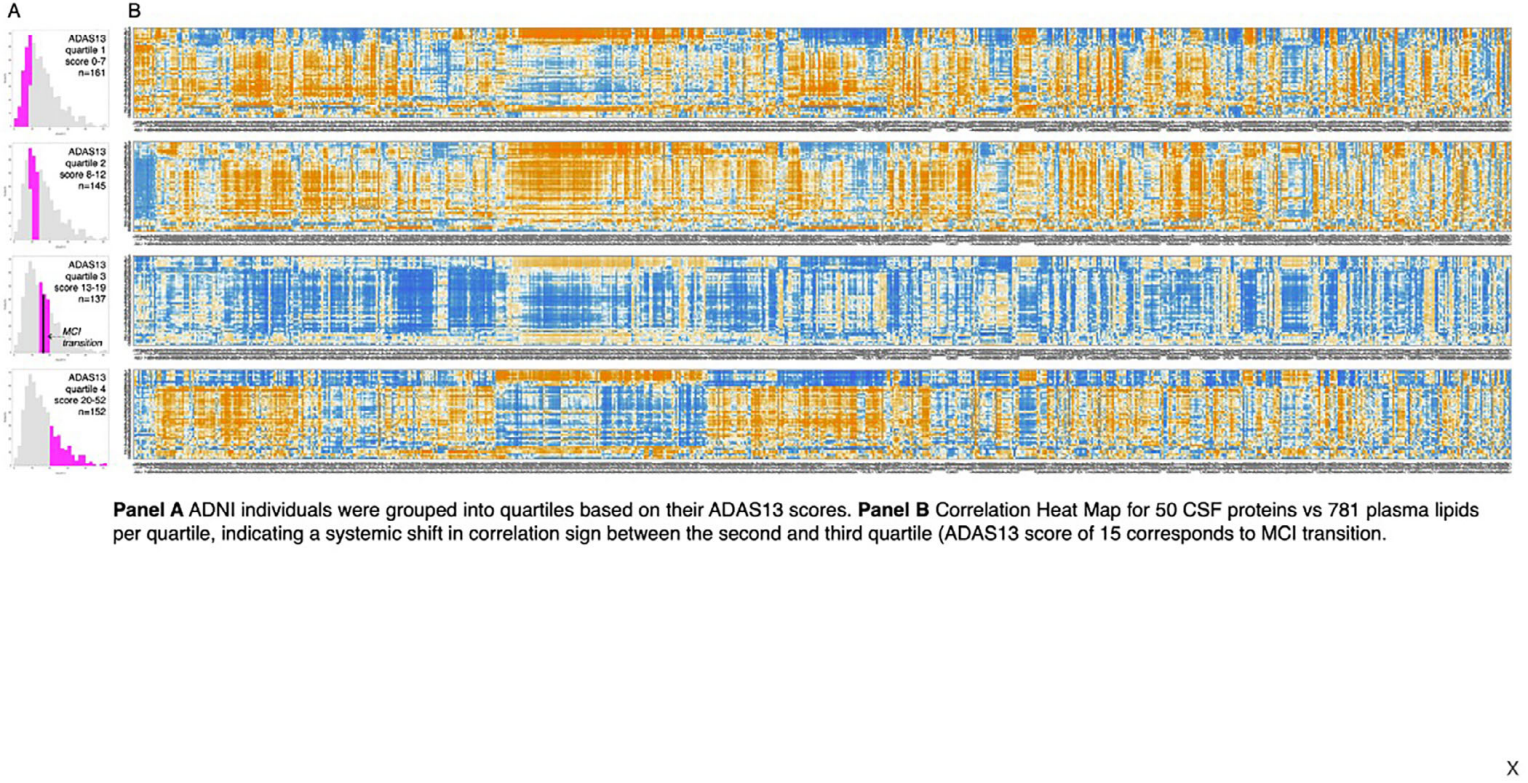# Analysis of ADNI phenotypic and mulit‐omic data identifies the role of microglial debris and lipid processing in Alzheimer’s Disease transition

**DOI:** 10.1002/alz.092897

**Published:** 2025-01-03

**Authors:** Cory C Funk, Tom Paterson, Jennifer Rohrs, Timothy J. Hohman

**Affiliations:** ^1^ Institute for Systems Biology, Seattle, WA USA; ^2^ EmbodyBio, Marin County, CA USA; ^3^ Department of Neurology, Vanderbilt University Medical Center, Nashville, TN USA

## Abstract

**Background:**

Microglial processing and recycling of debris is implicated in AD. AD GWAS loci are enriched for genes in efferocytosis, phagocytosis, endosomal trafficking and cholesterol efflux. Acting as a buffer, lipid droplets increase as a consequence of an imbalance between lipid debris influx and efflux rates. We hypothesize that a pivotal point in disease progression occurs when this buffering saturates, resulting in expanded lipid‐droplet accumulating microglia (LDAM). We anticipate a positive‐feedback effect where saturation accelerates neurodegeneration via inflammation and lipid processing via astrocytes. Susceptibility to this saturation may be connected to microglial AD GWAS loci—and would be reflected in progression rates. To explore this hypothesis, we analyzed lipidomic, proteomic, cognitive and genetic ADNI data to identify a signature indicative of this inflection point.

**Method:**

We identified ADNI individuals in comparable states of disease progression, grouping them into ADAS13 (cognitive score) quartiles, then examined blood lipidomic and CSF proteomic data. This resulted in a cross‐sectional dataset of 608 subjects spanning from cognitively normal to demented. We examined protein/lipid correlations across each quartile, identifying and characterizing inflection points.

**Result:**

We observed a stark and unique pattern of correlations in individuals with an ADAS13 score range from 13 to 19. We observed two subpopulations that differed in their rate of disease progression. CSF proteins in the rapid progressors point to microglial lipid droplet signaling and the lactate shuttle. We observed key changes in plasmalogens and cholesterol esters with DHA fatty acid tails, both indicative of neuronal debris processing and reverse cholesterol transport, as well as changes in acylcarnitines—indicative of reduced lipid hydrolysis. We also found differential enrichment of specific AD GWAS loci between the fast and slow progressors.

**Conclusion:**

Our analysis provides evidence for the importance of lipid droplet saturation coinciding with correlational changes in CSF proteins and plasma lipids, occurring in individuals transitioning from cognitively normal to MCI. We connected microglial AD GWAS loci to differences in the progression rates at this transition. These results have potential clinical relevance for predicting progression rate at MCI diagnosis, and inform our understanding of the role of GWAS loci in key transitions.